# Supplementary material for: Brd4 binds to active enhancers to control cell identity gene induction in adipogenesis and myogenesis
Source: Nat Commun. 2017 Dec 20;8:2217. doi: 10.1038/s41467-017-02403-5 (PMC5738375; doi:10.1038/s41467-017-02403-5)
Supplement: Supplementary file 1 — Supplementary Information [file 41467_2017_2403_MOESM1_ESM.pdf]

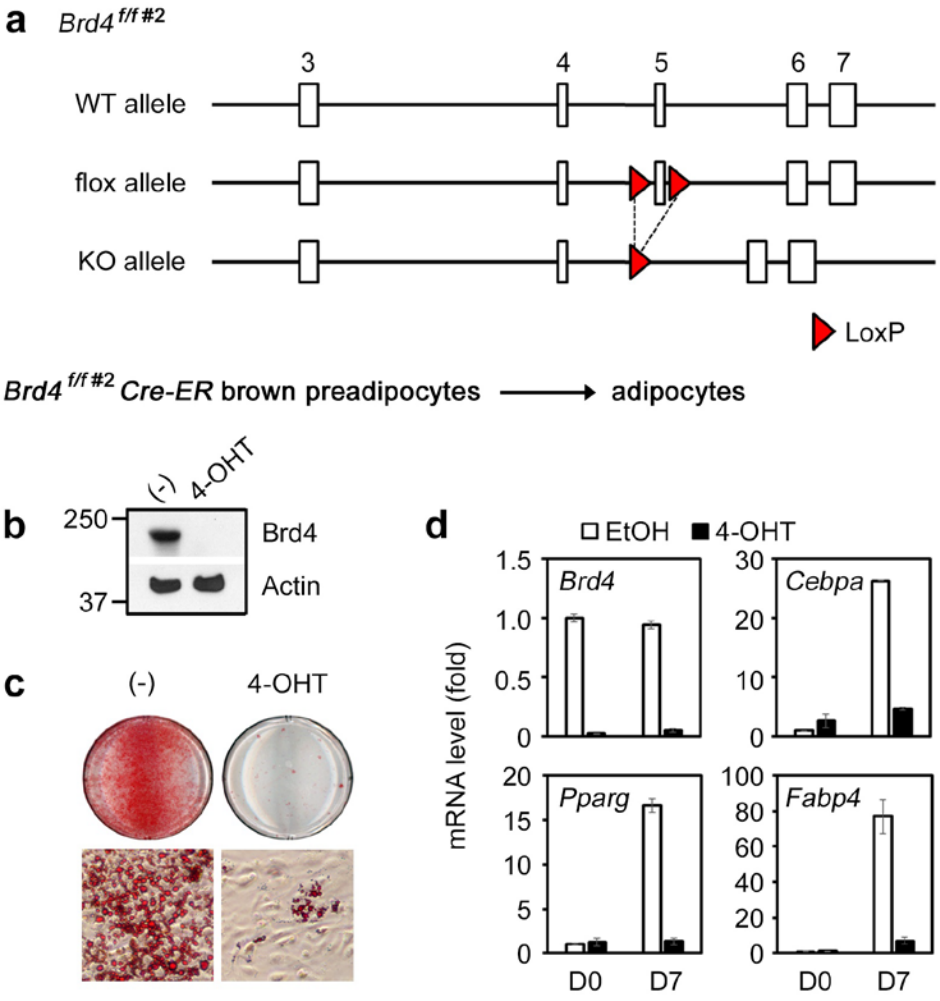

Supplementary Figure 1. Confirming the essential role of Brd4 in adipogenesis using cells derived from the second *Brd4* conditional KO mouse strain.

(a) Schematic representation of wild-type (WT), KO, and conditional KO (flox) alleles in the second *Brd4* conditional KO mouse strain *Brd4*$^{f/f\ \#2}$. In the flox allele, exon 5 was flanked by two loxP sites.

(b – d) Immortalized *Brd4*$^{f/f\ \#2}$;*Cre-ER* brown preadipocytes were treated with 4-hydroxytamoxifen (4-OHT) to induce the deletion of exon 5 of *Brd4* gene, followed by adipogenesis assay. (b) Deletion of Brd4 was confirmed by Western blot analysis. Actin was used as a loading control. (c) Oil red O staining at D7 of adipogenesis. Upper panels, stained dishes; lower panels, representative fields under microscope. (d) qRT-PCR of *Brd4*, *Pparg*, *Cebpa* and *Fabp4* expression at indicated time points during adipogenesis. Quantitative PCR data are presented as means ± SD.

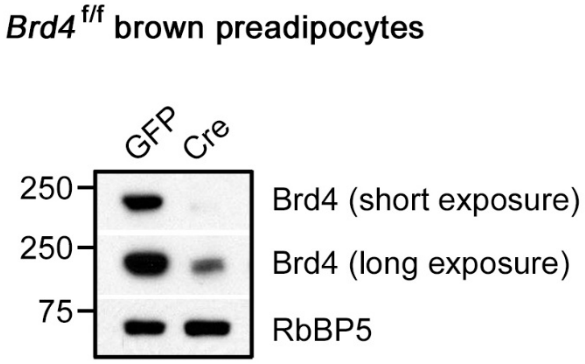

Supplementary Figure 2. Western blot of Brd4 in *Brd4*$^{f/f}$ brown preadipocytes.

In cells derived from the first *Brd4* conditional KO mouse strain *Brd4*$^{f/f}$, exon 3 was flanked by two loxP sites (see Figure 1a). Cre-mediated deletion of exon3 presumably allows translation from a downstream alternative start site in exon 4 and produces low levels of truncated protein (Δ1-104aa). Relative expression levels are shown by Western blot. The Brd4 (short exposure) and RbBP5 blots are also shown in Figure 2b.

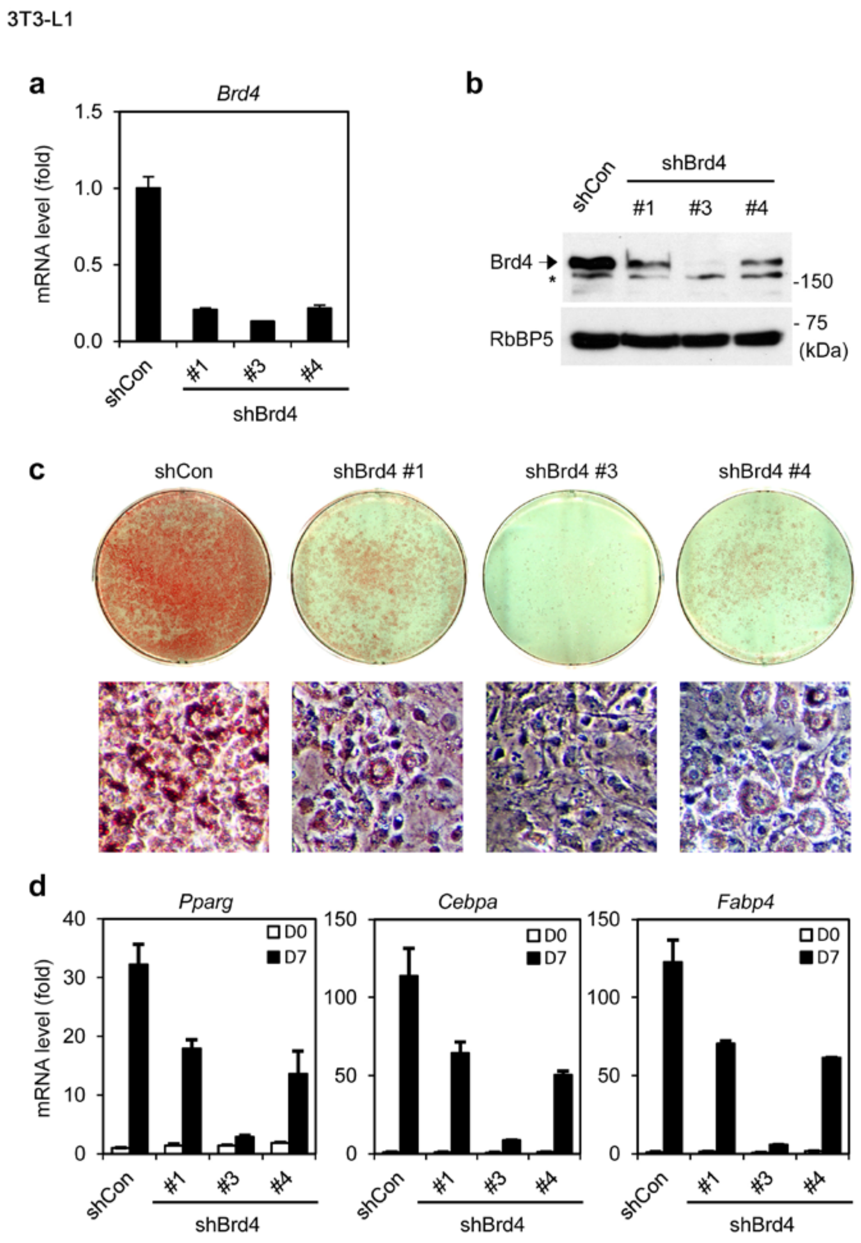

Supplementary Figure 3. Brd4 is required for 3T3-L1 adipogenesis.

3T3-L1 white preadipocytes were infected with lentivirus shRNAs targeting *Brd4* or control (Con) virus, followed by adipogenesis assay. (a) qRT-PCR confirmation of *Brd4* knockdown efficiency before adipogenesis. (b) Western blot analysis of Brd4 before adipogenesis. The asterisk indicates a non-specific band. (c) Oil red O staining at D7 of adipogenesis. (d) qRT-PCR of *Pparg*, *Cebpa* and *Fabp4* expression before (D0) and after (D7) adipogenesis. Quantitative PCR data are presented as means ± SD.

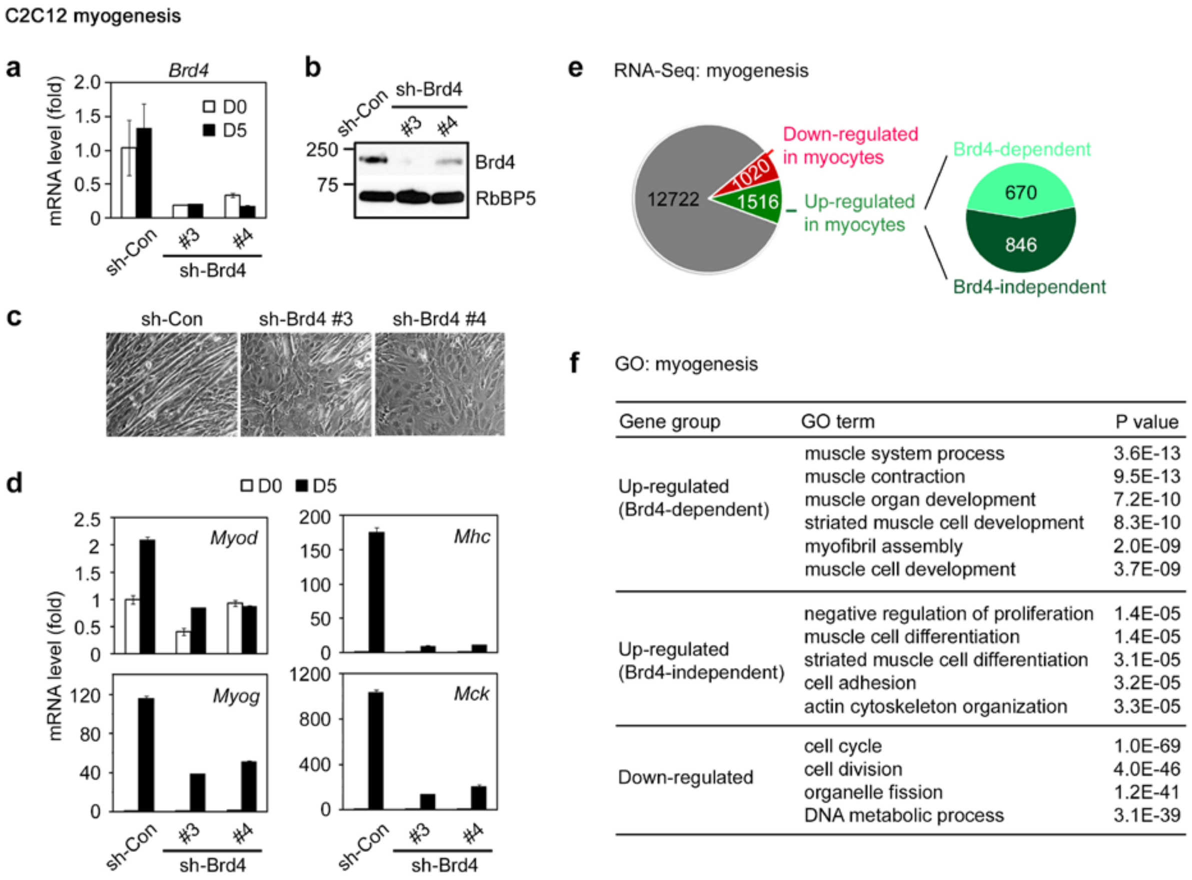

Gene group	GO term	P value
Up-regulated (Brd4-dependent)	muscle system process	3.6E-13
	muscle contraction	9.5E-13
	muscle organ development	7.2E-10
	striated muscle cell development	8.3E-10
	myofibril assembly	2.0E-09
	muscle cell development	3.7E-09
Up-regulated (Brd4-independent)	negative regulation of proliferation	1.4E-05
	muscle cell differentiation	1.4E-05
	striated muscle cell differentiation	3.1E-05
	cell adhesion	3.2E-05
	actin cytoskeleton organization	3.3E-05
Down-regulated	cell cycle	1.0E-69
	cell division	4.0E-46
	organelle fission	1.2E-41
	DNA metabolic process	3.1E-39

Supplementary Figure 4. Brd4 controls cell identity gene induction during myogenesis.

(a–d) Knockdown of *Brd4* inhibits myogenesis of C2C12 myoblasts. C2C12 cells were infected with lentivirus shRNAs targeting *Brd4* or control (Con) virus, followed by myogenesis assay. (a) qRT-PCR confirmation of *Brd4* knockdown efficiency before and after myogenesis. (b) Western blot analysis of Brd4 before myogenesis. (c) Cell morphology under microscope at D5 of myogenesis. (d) qRT-PCR analysis of myogenesis markers *Myod*, *Myog*, *Mhc*, and *Mck* expression before (D0) and after (D5) myogenesis. (e–f) Brd4 is required for induction of cell identity genes during myogenesis. (e) Schematic of identification of Brd4-dependent and -independent up-regulated genes during C2C12 myogenesis. The cut-off for up- or down-regulation is 2.5-fold. The cut-off for Brd4-dependency is 1.5-fold. (f) Gene ontology (GO) analysis of gene groups defined in (e). Quantitative PCR data are presented as means ± SD.

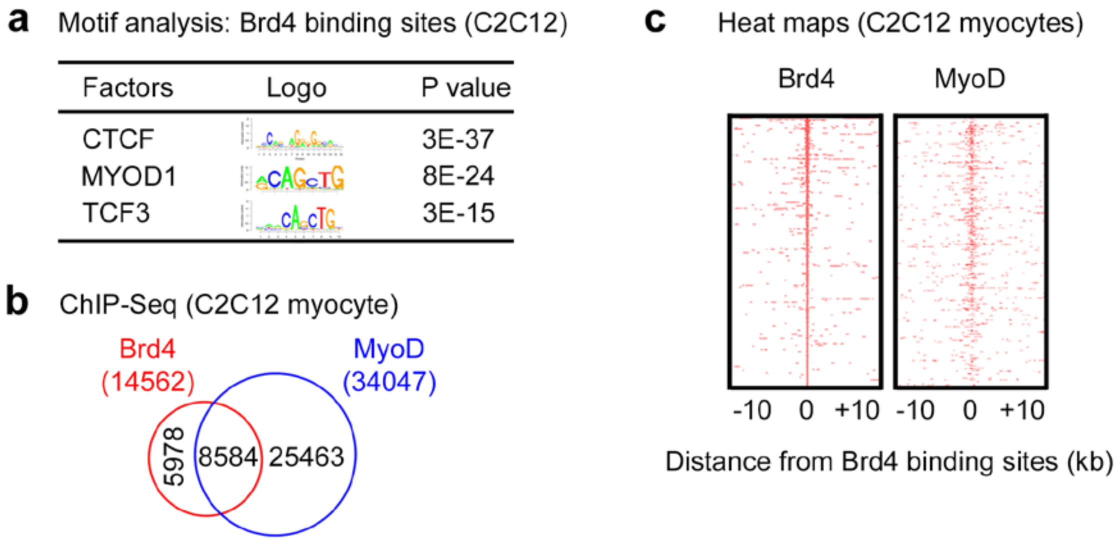

Supplementary Figure 5. Genomic co-localization of Brd4 with myogenic TF MyoD in myocytes.

A published ChIP-Seq data set for MyoD in C2C12 cells was used (GSE44824) [1].

(a) Motif analysis of Brd4 binding regions in C2C12 myocytes. (b) Venn diagram and (c) heat maps of genomic co-localization of Brd4 with MyoD in C2C12 myocytes.

a Motif analysis: Brd4^{+} AEs

Factors	P value		
	D0	D2	D7
JUN	3E-62		
JDP2	6E-51		
JUND	3E-27		
TCF3	2E-19		
HLF		3E-34	
GR		1E-15	
C/EBPα		<1E-307	2E-219
C/EBPβ		1E-307	1E-224
ATF4		8E-82	9E-72
NFIC		3E-53	2E-80
DBP		3E-50	2E-24
PPARγ		1E-40	2E-52
EBF1		3E-40	1E-40
PPARα			8E-43
ZFX			1E-26
E2F4			1E-17
CREB1			1E-16
TBX3			3E-16

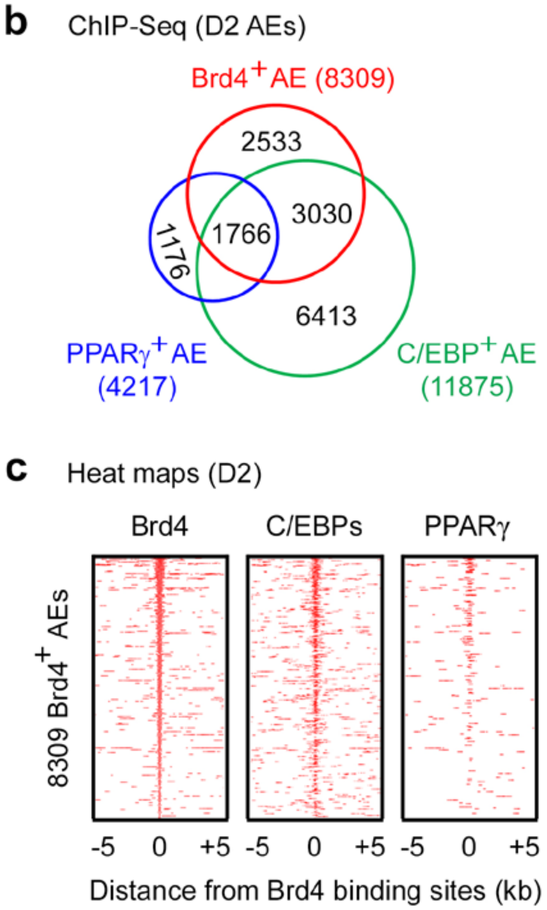

Supplementary Figure 6. Brd4 colocalizes with adipogenic TFs on active enhancers during adipogenesis.

(a) Motif analysis of Brd4^{+} active enhancers during adipogenesis. Top 3,000 Brd4 binding regions on active enhancers were used for motif analysis. Only TFs that are expressed at the indicated cell stages are shown. (b–c) Venn diagram (b) and heat maps (c) of Brd4, C/EBPs (C/EBPα or β) and PPARγ on active enhancers at D2 of adipogenesis.

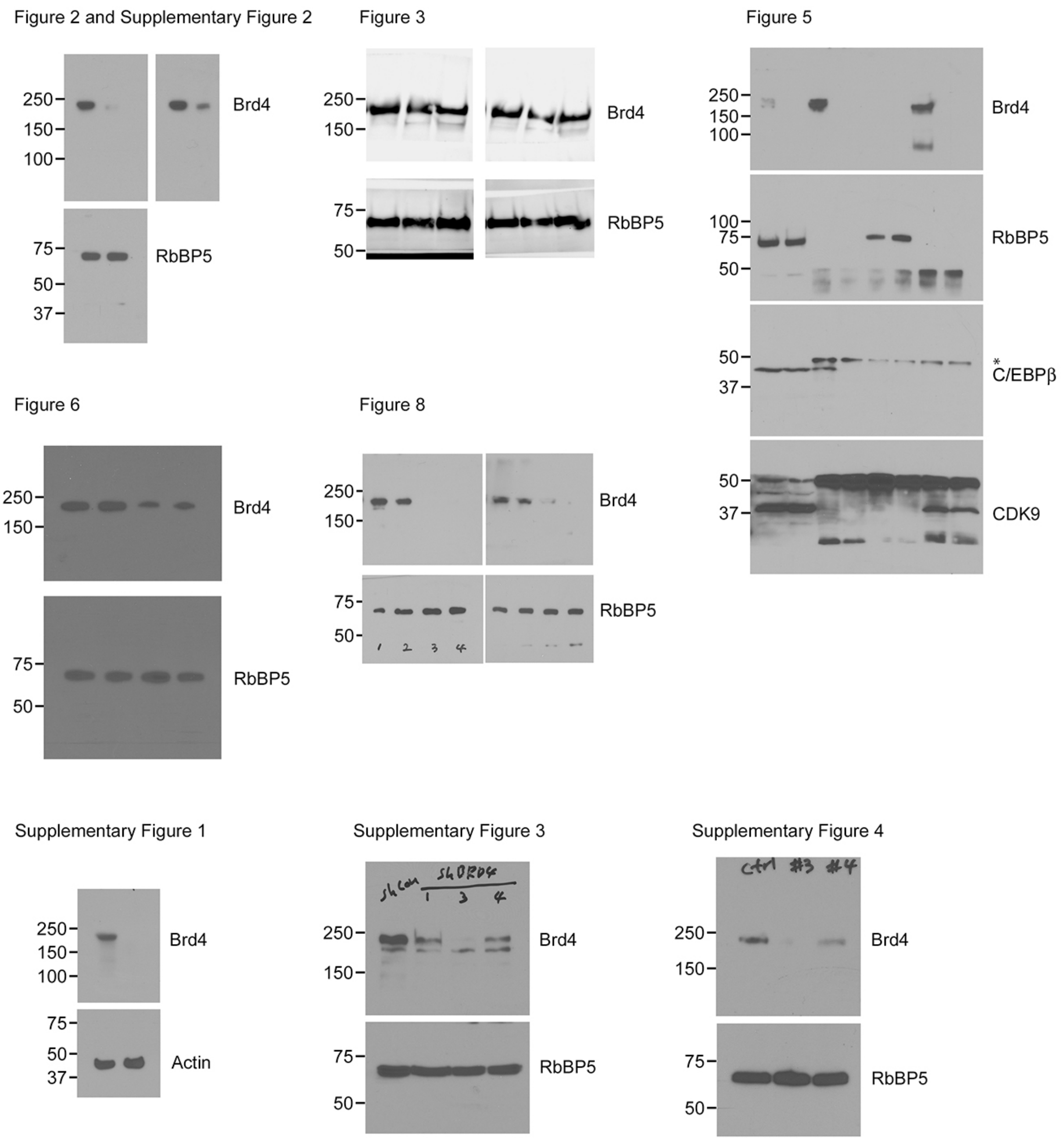

Supplementary Figure 7. Uncropped blots

Supplementary References

1 Marinov, G. K., Kundaje, A., Park, P. J. & Wold, B. J. Large-scale quality analysis of published ChIP-seq data. *G3* **4**, 209-223, doi:10.1534/g3.113.008680 (2014).
